# Lgr5 and stem/progenitor gene expression in gastric/gastroesophageal junction carcinoma – significance of potentially retained stemness

**DOI:** 10.1186/s12885-020-07362-7

**Published:** 2020-09-07

**Authors:** Ju-Yoon Yoon, Christine Brezden-Masley, Catherine J. Streutker

**Affiliations:** 1 Department of Laboratory Medicine and Pathobiology, University of Toronto, Toronto, Ontario Canada; 2 Department of Hematology/Oncology, Mount Sinai Hospital, Toronto, ON Canada; 3 Department of Pathology, St. Michael’s Hospital, St. Michael’s Hospital, Unity Health Toronto, Rm 2-099CC, 30 Bond Street, Toronto, Ontario M5B-1W8 Canada

**Keywords:** Lgr5, Gastric/gastroesophageal junction carcinoma, Stem/progenitor cell

## Abstract

**Background:**

Gastric/gastroesophageal junction (GEJ) adenocarcinomas are heterogeneous, comprising four molecularly distinct subtypes, namely EBV-positive, microsatellite instability (MSI), chromosomal instability (CIN) and genomically stable (GS) subtypes, and a part of this heterogeneity may hypothesized to be different cells-of-origin. Stem/progenitor cell hierarchy in the stomach is complex, which include the Lgr5^(+)^ gastric stem cells (GSCs).

**Methods:**

While previous studies have focused on non-nuclear Lgr5 expression, nuclear Lgr5 expression has been reported in a subset of stem cells, and we examined nuclear Lgr5 expression in a local cohort of 95 cases of gastric/GEJ adenocarcinoma. mRNA levels for *LGR5* and other stem cell marker genes were examined in the TCGA cohort.

**Results:**

We observed nuclear Lgr5 expression in a 18/95 cases. Near mutual exclusivity was seen between nuclear Lgr5 and strong non-nuclear Lgr5. Both strong non-nuclear and nuclear Lgr5 expression tended to be seen more frequently with the intestinal histotype and approximated CIN molecular subtype. With respect to overall survival (OS), nuclear Lgr5 expression appears to be protective, with the worst survival being seen in the cases lacking nuclear Lgr5 and with low non-nuclear Lgr5 expression. When compared to other stem/progenitor cell markers, *LGR5* mRNA expression clusters with other GSC marker genes, including *VIL1*. Higher expression of these GSC marker genes was associated with better OS.

**Conclusions:**

Our results show that Lgr5 expression is dynamic in gastric/GEJ adenocarcinoma and heterogeneous across the several disease attributes. We postulate that this may reflect “retained stemness” in the form of Lgr5^High^-GSC signature that appears to be associated with better survival.

## Synopsis

Significance of Lgr5 expression in gastric/GE junction carcinomas is re-examined in the context of their molecular heterogeneity. Both nuclear and non-nuclear Lgr5 tended to be associated with the intestinal histotype, chromosome instability molecular subtype. *LGR5* mRNA expression cluster was associated with better prognosis. High Lgr5/*LGR5* expression is speculated to be a retained phenotype that may be reflecting the cellular origin in a subset of gastric carcinomas.

## Background

Gastric/gastro-esophageal junction (GEJ) adenocarcinomas comprise a morphologically, molecularly and clinically diverse group of cancers. TCGA studies demonstrated that gastric/GEJ carcinomas are a heterogeneous group of diseases that could be sub-categorized into four molecular subtypes, namely EBV-related, microsatellite instability (MSI), chromosomal instability (CIN) and genomically stable (GS) [1, 2]. While the molecular heterogeneity to some extent reflects the different etiologies and pathways of tumorigenesis, such as deficiency in the mismatch repair process (leading to the MSI subtype tumors), another potential factor contributing to the molecular diversity in gastric/GEJ adenocarcinoma is the poorly explored concept of “cell-of-origin”.

Applying the cell-of-origin concept to gastric/GEJ adenocarcinoma pathogenesis is challenging, related as it is to the complex biology of stem/progenitor cells in the stomach. The gastric mucosa is a site of dynamic regenerative homeostasis, with turnover that is thought to be maintained by long-lived stem/precursor cells, with a number of studies that point to Lgr5^(+)^ cells as the self-renewing, multipotent stem cells [3, 4]. The key signaling pathway behind this phenomenon is thought to be the canonical Wnt pathway, with Lgr5 functioning to potentiate Wnt/β-catenin signalling by interacting with R-spondin, among other factors [5, 6]. This role of Lgr5 is presumably facilitated by its trans-membrane domain allowing for membranous localization, but Lgr5 is a spatially dynamic protein, with expression seen also in the cytoplasm and even the nucleus [7–9]. Surface Lgr5 expression is partially regulated by endocytosis, and inhibiting clathrin-mediated endocytosis of Lgr5 diminishes intestinal epithelial cell fitness in murine models [8]. In the correct in vitro conditions, isolated single Lgr5^(+)^ stem cells have the remarkable capacity to form organoids, known as “mini-guts” [3]. Long-term ablation of Lgr5^(+)^ cells impairs epithelial homeostasis in the corpus [10]. In homeostasis, the majority of the Lgr5^(+)^ stem cells appear to divide symmetrically [11], and their proliferation can be accelerated by *H. pylori* infection [12]; this may represent a potential link between the epidemiological observation of increased risk of carcinogenesis associated with *H. pylori* infection. Lgr5 has also been postulated to be a marker for cancer stem cells (CSCs) in gastric cancer [10, 13], although the hypothesis is currently lacking in definitive experimental results. While concepts may be overlapping, CSCs differ from cells-of-origin in that CSCs generally refer to progenitor/stem-like cells isolated from cancer specimens, generally with ability to form tumors in xenograft models [14]. CSCs that have been obtained and characterized from the stomach have been isolated using a number of different mesenchymal stem cell (MSC) markers such as CD90 [15–17]. Interestingly, more primitive MSC/MSC-like cells are also found in normal stomach, and some studies have attributed roles in intestinal and gastric homeostasis to these MSC/MSC-like cells [16].

When analyzed agnostic to the intrinsic heterogeneity in gastric/GEJ adenocarcinoma, Lgr5 expression does not carry a statistically significant prognostic weight, although some prognostic significance is seen in early stage (I/II) patients [18, 19]. Studies have associated higher Lgr5 expression with more frequent nuclear β-catenin expression, a marker of active Wnt signalling, and higher Lgr5 has been associated with worse survival in a subset of patients. However, these studies predated the TCGA molecular subtyping, and cellular sub-localization of Lgr5 is now better characterized; nuclear Lgr5 has been reported in hair follicle stem cells, and their expression was observed to be limited to cycling hair follicle stem cells, after asymmetric self-renewal division [7]. In light of these previous reports and better understanding of Lgr5 biology, we examined the prognostic value of *LGR5* mRNA and Lgr5 protein, including its nuclear expression, and we frame the discussion to focus on the potential implication in histogenesis.

## Methods

### Tissue microarray and immunohistochemistry

This study was performed in conjunction with our institution’s research ethics board (REB 10–280). We studied a cohort that has been described in our previous reports [20, 21], where we identified cases of gastric/GEJ carcinoma patients treated at the St. Michael’s Hospital (SMH, Toronto, Ontario, Canada) with either gastrectomy or endoscopic mucosal resection, between the period 2001 to 2011. A tissue microarray (TMA) was constructed as described previously [20], consisting of two 0.6 mm cores per each tumour, with several corresponding normal cores. Histology subtypes were obtained from the pathology reports associated with each case, and diffuse histology was interpreted as per the Lauren classification. Any cases with mixed histology were categorized as “other”.

Lgr5 immunohistochemistry was performed using a rabbit monoclonal antibody, clone EPR3065Y (Abcam, catalogue number Ab75850), with 1/100 dilution, incubated overnight. Binding of the primary antibody was subsequently detected using ImmPRESS Anti-Rabbit IgG (Vector, catalogue number MP − 7401) and the diaminobenzidine substrate kit (DAKO, catalogue number K3468). Non-nuclear Lgr5 staining intensity and frequency was scored by applying the Allred method [22]. Briefly, the method combines staining intensity (0–3) with staining frequency (0–5, with 5 representing > 66% of cells staining), for maximum score of 8. The method was modified to examine cytoplasmic/membranous (vs. nuclear) staining, and the scores were converted to “low” (Allred score ≤ 6), and “high” (> 6).

### Approximation of the molecular subtypes

We had previously described our method for approximation of the molecular subtype [21]. Briefly, we employed a subtyping algorithm based on the TCGA algorithm, a series of dichotomizing steps. We first identified the EBV-CIMP cases, identified by EBER in-situ hybridization positivity. The MSI subtypes were next identified through immunohistochemistry (IHC) for mismatch repair (MMR) pathway proteins, MLH1, PMS2, MSH2 and MSH6. Among the remaining MMR intact, EBER-negative cases, GS and CIN subtypes were distinguished based on the histotypes (diffuse vs. Intestinal/mixed, respectively).

### Identification of and analysis of stem/progenitor cell marker genes

A list of nineteen genes were generated through literature review, starting with review papers on gastric stem cells and cancer stem cells in gastric cancer [15, 23]. Some markers were excluded due to lack of interpretable values from the TCGA mRNA levels (z-score values, RNA Seq V2) (ex. *CD24*, *BMI1*). SSEA-3 and SSEA-4 are non-protein markers and were substituted with *ST3GAL2*, which encodes the enzyme responsible for conversion of SSEA-3 to SSEA-4 [24]. The TCGA data were downloaded from cBioPortal (http://www.cbioportal.org/) in the form of mRNA z-score values (RNA Seq V2 RSEM), restricting to the cases that were part of the 2014 TCGA publication [2]. All clinical and pathological data for the TCGA cohort were downloaded from cBioPortal.

### Clustering and statistics

Unsupervised clustering of mRNA expression levels/patterns were performed using Cluster 3.0, and visualized using TreeView [25]. The obtained hierarchical clustering results were used in grouping the TCGA cohort into groups A-C. Survival analysis was performed using the Kaplan-Meier method. Comparisons of continuous variables between multiple groups were performed using analysis of variance (with Tukey post hoc tests). Comparisons of categorical variables between multiple groups were performed using Chi-square test. All statistical tests were performed using JMP (SAS version 13/14).

## Results

### Lgr5 expression patterns in gastric/GEJ carcinoma

**Fig. 1:**
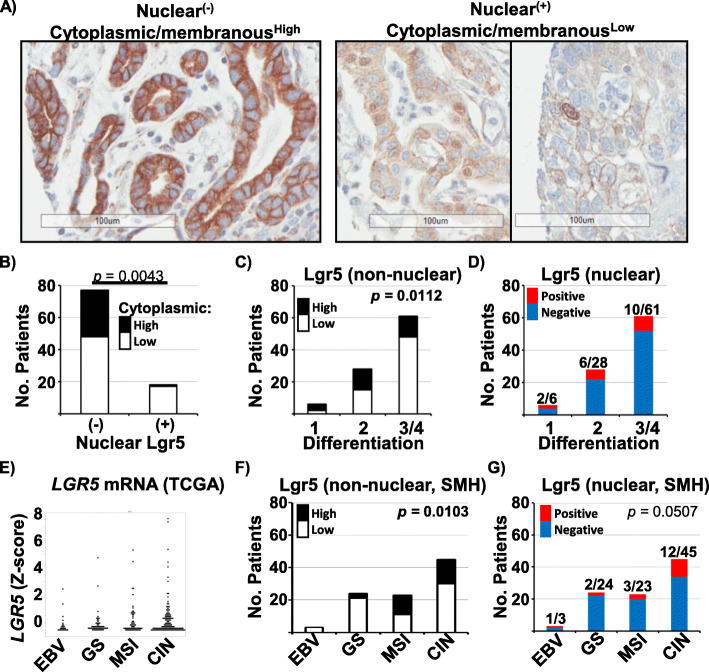
**a** Representative IHC sections, showing nuclear and cytoplasmic/membranous Lgr5 staining. Bar = 100 μ. **b** Relationship between nuclear and non-nuclear (cytoplasmic and membranous) Lgr5 levels in the SMH TMA cohort. **c** Non-nuclear Lgr5 expression with respect to tumor differentiation scores. **d** Nuclear Lgr5 intensity with respect to differentiation. **e** Comparison of *LGR5* mRNA levels amongst the indicated TCGA molecular subtypes in the TCGA cohort. **f** and **g** Comparison of non-nuclear (**f**) and nuclear (**g**) Lgr5 protein expression levels amongst the approximate molecular subtypes in the SMH cohort. Statistically significant *p* values are indicated

We examined Lgr5 protein expression in our previously described Toronto (St. Michael’s Hospital) TMA cohort of gastric adenocarcinoma cases [20, 21]. Non-nuclear (cytoplasmic/membranous) staining intensity was variable, ranging from being completely barely perceptible, low staining (low) to diffuse, strong positivity (high) (Fig. 1a, left). Careful examination of the cores showed that nuclear Lgr5 expression can be seen in 18/95 cases of gastric/GEJ adenocarcinoma (Fig. 1a, right). Areas could be identified showing clear overlap between nuclear Lgr5 expression and frank nuclear atypia, indicating nuclear expression by carcinoma cells (vs. entrapped non-tumour cells). Nuclear staining distribution was relatively bimodal, being either none/very focal (less than 1% of lesional cells, i.e. negative) or patchy, weak-to-moderate expression in more than 1% of lesional cells (i.e. positive). Cases with high non-nuclear staining generally lacked nuclear Lgr5 staining (*p =* 0.0043) (Fig. 1b). Interestingly, one case with high non-nuclear Lgr5 and nuclear Lgr5 comprised a TMA core containing both better differentiated (gland-forming) areas with high non-nuclear Lgr5 and another area with nuclear Lgr5 seen in the more solid, higher grade area (Supp. Figure 1).

In gastric adenocarcinoma, pathologists assess “differentiation”, based on the lesion’s resemblance to normal stomach histology with respect to the proportion of the tumor forming well-organized glandular structures, ranging from 1 (well-differentiated) to 4 (undifferentiated). When Lgr5 expression was examined across the differentiation spectrum, high cytoplasmic/membranous staining was seen more frequently with well-to-moderately differentiated (i.e. grade 1, 2), compared to poorly to undifferentiated (i.e. grade 3, 4) (*p =* 0.0112, Fig. 1c), and these results are in line with the previously reported observation [18]. Similarly, nuclear Lgr5 positivity was seen more frequently with better differentiated cancers (8/34 in grade 1–2 vs. 10/61 in grade 3–4), but the difference was not statistically significant (Fig. 1d). A related concept is histotyping of gastric adenocarcinoma; by Lauren classification, gastric adenocarcinoma is divided into intestinal, diffuse and mixed types [26]. In the TCGA cohort, the largest range for *LGR5* mRNA levels was observed in the intestinal group, but differences were not statistically significant (Supp. Figure 2). In the SMH cohort, Lgr5 protein expression (both nuclear and non-nuclear) was seen most frequently in the intestinal histotype, and the difference approached statistical significance (*p* = 0.0507).

We next examined Lgr5 protein/*LGR5* mRNA across the different molecular subtypes. In the TCGA cohort, EBV subtype expressed the lowest levels of *LGR5* mRNA, with one comparison (CIN vs. EBV subtypes) being statistical significant (*p =* 0.0451) (Fig. 1e). Examining the protein levels in the SMH cohort by approximated molecular subtypes, cases with strong cytoplasmic Lgr5 were most frequently seen with the CIN and MSI subtypes (*p* = 0.0103) (Fig. 1f). Most of the cases with nuclear Lgr5 expression (12/18 cases with positive nuclear expression) were of the CIN subtype (Fig. 1g), but this difference did not reach statistical significance.

### Stem/progenitor cell marker mRNA expression patterns and different subtypes of gastric/GEJ carcinoma

**Fig. 2:**
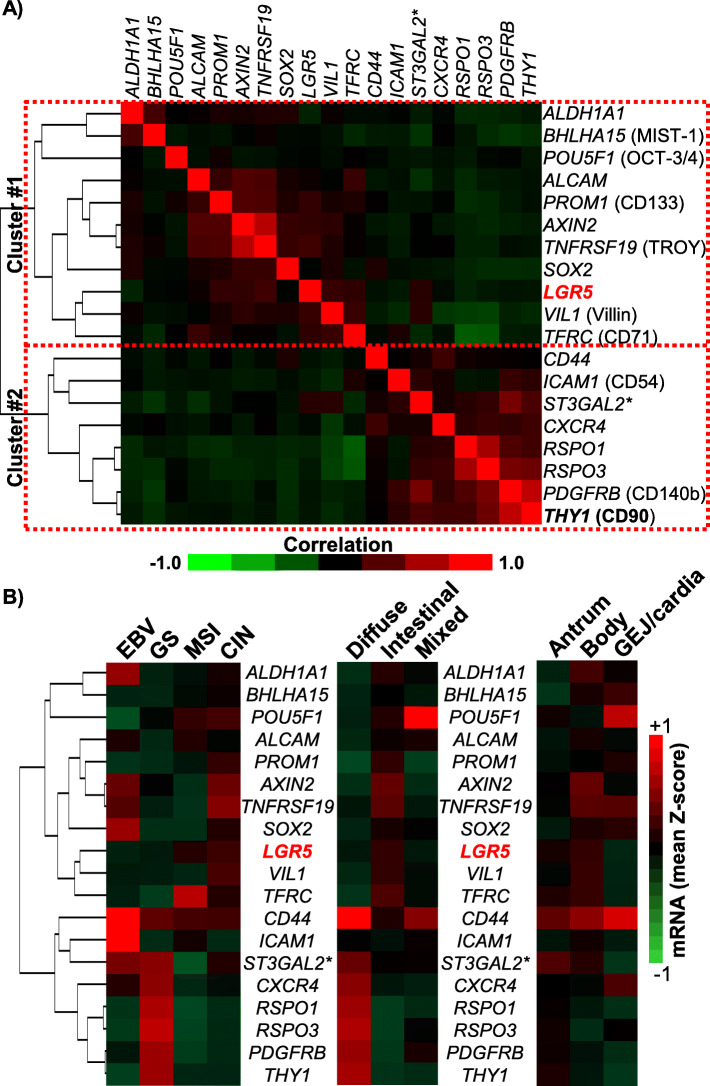
Stem/progenitor cell marker expression pattern and different subtypes of gastric/GEJ carcinoma. **a** Clustered correlation heatmap, displaying the correlation coefficients between the nineteen different stem/progenitor cell markers. **b** Mean expression levels (Z-score values) for the nineteen stem/progenitor cell markers in the different molecular subtypes (left), histotypes (center) and anatomical tumor epicenter (right)

The stomach is home to several distinct stem/progenitor cell populations, with Lgr5 marking just one of the sub-populations. A number of markers have been used to sort and trace these gastric stem cells (GSCs), as well as cancer stem cells (CSCs) from gastric cancer samples [15, 23]. Nineteen stem/progenitor cell marker genes were selected based on literature review and TCGA expression data availability (see methods). Examining the mRNA levels in the TCGA cohort, correlation clustering showed two groups, 1) *LGR5*-containing cluster (cluster #1), which included a number of other well-established gastric stem cell (GSC) markers, such as *SOX2* and *VIL1*, and 2) *THY1* (CD90)-containing cluster (cluster #2), which contained a number of genes that have been established as either mesenchymal stem cell (MSC) markers and/or CSC markers, including *CD44*, *PDGFRB* (encoding CD140b), as well as more recently characterized GSC markers, *RSPO1* and *RSPO3* (Fig. 2a). The strongest positive correlations with *LGR5* mRNA levels were seen with *TNFRSF19* (encoding TROY, Pearson correlation coefficient (PCC) = 0.2517, *p* < 0.0001) and *VIL1* (encoding Villin, PCC = 0.2185, *p* = 0.0003). The strongest positive correlations with *THY1* were with *PDGFRB* (PCC = 0.7482, *p* < 0.0001*)* and *RSPO3* (PCC = 0.425, *p* < 0.0001).

We next examined the mRNA levels of the same nineteen genes across different molecular subtypes and histological subtypes in the TCGA cohort (Fig. 2b). As expected from the mRNA clustering patterns, the subtypes with higher expression levels of cluster #1 genes (containing *LGR5*) tended to express lower levels of cluster #2 genes (containing *THY1*). In particular, the CIN molecular subtype and intestinal histotypes were notable for highest expression levels of the cluster #1 genes. Cluster #2 genes were expressed at higher levels in the GS molecular subtype and diffuse histotypes.

The stomach is divided into different anatomical regions, namely the GEJ, body/fundus and the antrum/pylorus, and the incidence of different molecular subtypes differ based on the anatomic location within the stomach (Supp. Figure 3A and [1, 2]). When we compared the TCGA cohort by their anatomical origin, the cluster #1 genes tended to be expressed at higher levels in the fundus/body tumors compared to those from other regions, while higher cluster #2 gene expression in the antral tumors (Fig. 2b, right). Focusing on *LGR5*, fundus/body and antral tumors expressed higher levels of *LGR5* mRNA, and the difference was largely driven by CIN tumors (Supp. Figure 3).

### Patient survival and stem/progenitor marker expression

**Fig. 3:**
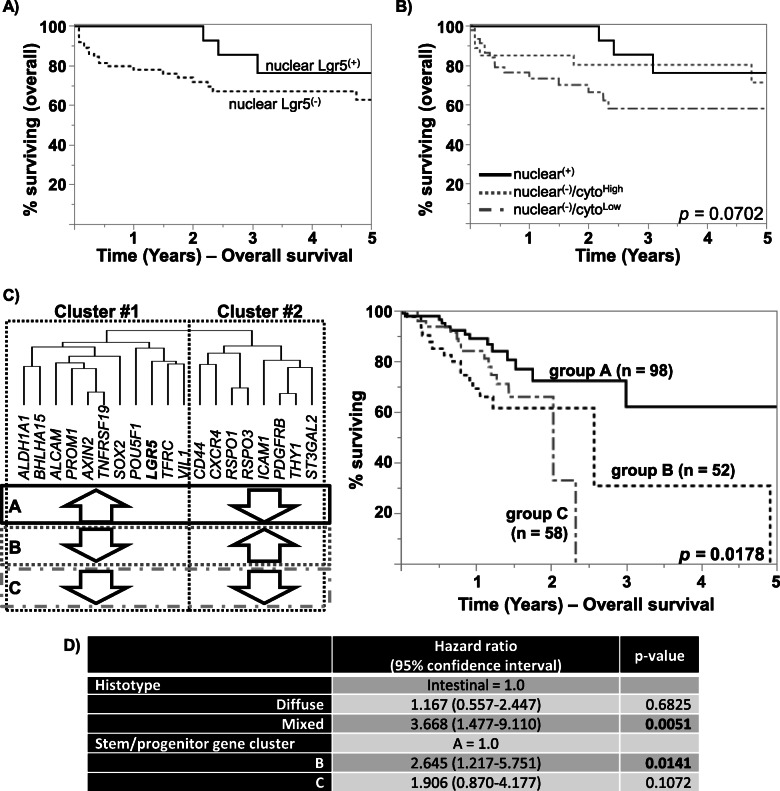
Kaplan-Meier survival curves showing overall survival in the SMH gastric/GEJ carcinoma cohort, grouped by (**a**) nuclear Lgr5 levels (log-rank *p* = 0.1742), and (**b**) combined cytoplasmic/membranous and nuclear levels combined (log-rank *p =* 0.0702). **c** The TCGA cohort was stratified based on the expression pattern of the indicated stem/progenitor cell marker genes. Overall survival in the TCGA cohort, divided into three groups by gene expression pattern of the stem/progenitor genes

When we examined cytoplasmic/membranous Lgr5 for its prognostic significance, there was no statistically significant difference in overall survival (OS) in our cohort (Supp. Fig. 4), in line with previous studies. By nuclear Lgr5 expression, patients with positive nuclear expression tended to have better OS (Fig. 3a). However, while the number of deaths were markedly different (3/18 for nuclear Lgr5^(+)^ vs. 21/77 for nuclear Lgr5^(−)^), the difference was not statistically significant (log-rank *p* = 0.1742). Noting the relationship between nuclear and cytoplasmic/membranous Lgr5, we next combined the nuclear and cytoplasmic Lgr5 patterns. The worst survival was seen in the cases lacking nuclear Lgr5, with low cytoplasmic/membranous staining (15/48 deaths vs. 9/47 in others), and this difference trended towards statistical significance (log-rank *p* = 0.0702) (Fig. 3b).

Noting the heterogeneous combination of expression patterns and the two clusters that emerged for stem/progenitor marker genes, we clustered the TCGA cohort based on the expression levels of the nineteen genes. The clustering resulted in 3 groups of cases: A) high expression level of cluster #1 genes (*n* = 117), B) high expression level of cluster #2 genes (*n* = 61), and C) low expression of both cluster #1 and #2 (*n* = 87) (Fig. 3c). As expected from our results above, group A contained the most number of CIN tumours (69/117), while group B contained the highest number of GS tumours (27/61). Examining patients with known survival data, best OS was seen with group A (median survival 69 months), compared with groups B (median survival 30.9 months) and group C (median survival 24.3 months) (log-rank *p =* 0.0178) (Fig. 3b). The differences between the survival curves are significant, considering the lack of survival difference by molecular subtypes in the TCGA study [2].

Taken together, gastric/GEJ carcinomas exhibit heterogeneous expression of the different stem/progenitor marker genes. Among them, *LGR* mRNA and Lgr5 protein expression pattern is dynamic, with respect to cellular localization (nuclear vs. cytoplasmic/membranous) and to tumor pathologic attributes (anatomical epicenter, histotype and molecular subtypes). Higher expression of GSC markers, including *LGR5* and *VIL1*, are seen with CIN/intestinal subtypes and associated with better survival. This is in contrast to many prior studies that have linked stem cell signatures with worse survival.

## Discussion

The general dogma in many studies that have examined “stemness” posit that stemness is associated with enhanced ability to invade, and thus ultimately being associated with worse survival. However, our data with Lgr5*/LGR5* suggest that this story is likely more complex—it depends on what stem signature is present. The immunohistochemical and mRNA expression profile phenotype with Lgr5^High^-GSC signature combination, which may represent “retained stemness”, was associated with better survival (Fig. 3). If this is indeed a phenotype retained from its cell-of-origin, we may speculate that these tumors retained their intrinsic predisposition for glandular differentiation, explaining the association seen with better differentiated, intestinal histo-morphology, and Lgr5 expression. Lgr5^(+)^ cells have been postulated to be the cellular origin of invasive gastric carcinoma by others, specifically in the intestinal histotype [27, 28]. Noting the association with the CIN molecular subtype, the Lgr5^(+)^ may have an intrinsic predilection for a specific tumorigenic pathway. Targeted deletion of *Smad4* and *PTEN* in Lgr5^(+)^ gastric stem cells in mice resulted in formation of invasive adenocarcinoma, whereas the same set of genetic maneuvers failed to induce tumorigenesis in differentiated cells, including antral parietal cells, pit cells, as well as corpus Lgr5^(+)^ chief cells [28]. The likely candidate precursor in this pathway would be intestinal metaplasia (IM), and both *Lgr5* mRNA and Lgr5 protein expression has been reported in IM [27, 29, 30]. Lgr5^(+)^ cells are seen at the crypt base in IM, and the expression becomes more marked in carcinoma in its luminal surface, tumor center and the invasion front type [10, 27]. Interestingly, while spasmolytic polypeptide-expressing metaplasia (SPEM), another putative precursor to gastric adenocarcinoma, was suggested to be not associated with Lgr5^(+)^ cells in the variegated Lgr5-EGFP-IRES-Cre (ERT2/+); in Rosa26R mice [31], expression of constitutively active *Kras* (harboring p.G12D hotspot mutation) in Lgr5^(+)^ chief cells was sufficient form SPEM in a non-variegated Lgr5-2A-CreERT2 mouse model [10]. Thus, at least two different pathogenic pathways involving Lgr5-expressing cells appear to be possible, both of which may give rise to CIN molecular subtype tumors. Interestingly, while the tumors with GEJ/cardia epicenter are largely of the CIN molecular subtype [1], these tumors expressed lower levels of *LGR5* mRNA. The abundance of Lgr5^(+)^ cells varies across the different anatomical regions, being most abundant in the antrum [3, 29]. Thus, there may also be heterogeneity amongst the CIN tumors, with those cardiac/GEJ tumors potentially arising from Lgr5-negative cells, perhaps including the recently described osteopontin responsive, Lgr5-negative/CD44^(+)^ cells [32]. In line with this possibility, GEJ/cardia tumors were notable for high *CD44* mRNA, although *CD44* mRNAs are likely to be expressed by a variety of non-tumoral cells, especially lymphocytes.

A part of the challenge in analyzing Lgr5 and its clinical significance is related to its complex biology, including its heterogeneous sub-cellular localization. The physiologic role of nuclear Lgr5 has only thus far been described in the hair follicle stem cells, where nuclear Lgr5 expression is limited to the cycling stem cell sister after asymmetric self-renewal divisions [7]. While the role of nuclear Lgr5 in the gastric epithelium and in cancer cells remains largely unexplored, it is worth noting that several studies examining Lgr5^(+)^ cells based their identification of Lgr5^(+)^ cells based on *LGR5* mRNA expression and/or *LGR5*-driven reporters (ex. *Lgr5*-lacZ), where the sub-cellular localization of reporters may differ from that of the endogenous Lgr5 [3, 10, 29]. Inferring from studies on hair follicle stem cells [7], nuclear Lgr5 may be highlighting the cancer cells with a yet a different pattern of “stemness”. While nuclear Lgr5 was nearly mutually exclusive with non-nuclear, strong Lgr5 expression, nuclear Lgr5 also showed similar associations with the intestinal histotype and CIN molecular subtype, suggesting nuclear Lgr5 may also represent a pattern of “retained” phenotype, similar to non-nuclear Lgr5.

Beside the Lgr5^(+)^/*LGR5*-expressing cells, more primitive MSC/MSC-like cells appear to be playing a crucial role in intestinal homeostasis, and MSC-like cells have been isolated from human gastric cancer tissues, marked by high expression of *THY1* [16, 17]. Some studies have even implicated bone marrow-derived MSCs in gastric epithelium homeostasis, which have been shown to repopulate the gastric epithelium [33, 34]. Interestingly, bone marrow-derived MSCs, at least in mouse models, appear to be able to give rise to both the carcinoma cells and the cancer-associated stromal cells, contributing to disease progression [33, 34]. In the context of these theories of gastric cancer carcinogenesis, the clustering pattern of different stem/progenitor cell markers seen in the TCGA data is interesting (Fig. 2a), which suggest that there may be at least two different signatures of stem/progenitor gene expression, i.e. one more intestinal (cluster #1, *LGR5*-high) and one more MSC-like (cluster #2, *THY1*-high). However, this is likely to be a simplified version of the story, considering the complex hierarchy between the different stem/progenitor cells in the GI tract [35, 36]. Future studies may thus benefit from examining co-expression patterns of multiple markers, including Bmi1, which appear to mark slow-cycling stem/progenitor cells that can revert to express Lgr5 [35, 37, 38], while noting the different sub-cellular Lgr5 expression patterns. One peculiar finding was the co-clustering of *RSPO1* and *RSPO3* in cluster #2. The mRNA levels of the two genes correlated highly with one another, while correlating little or negatively with the cluster #1 genes (Fig. 2a). R-spondins are ligands to Lgr5, and the “Rspo-Lgr5 axis” plays an important role in stimualting the growth of intestinal crypts in vitro via the Wnt pathway [39, 40]. While the “Rspo-Lgr5 axis” had been previously postulated to be merely an amplification process for Wnt signaling, more recent studies point to non-overlapping, non-interchangeable roles between Wnt and R-spondin in GSCs [41]. As well, R-spondins, at least R-spondin 3, can be produced by myofibroblasts in the stomach [6]. As fibroblasts/myofibroblasts are also marked by CD90 (Thy1) expression, these observations may be merely indicating enrichment of stromal cells. Alternatively, Lgr5, perhaps in its nuclear form, may be carrying out functions independent of R-spondins in gastric/GEJ carcinoma, and further studies would be required to better understand the Rspo-Lgr5 axis in gastric/GEJ carcinoma.

We encountered several challenges in this study, one of which was related to our relatively small cohort size, which likely explains the lack of statistical significance with some of the analyses (ex. OS analysis in Fig. 3a/b). Another weakness of this study is its purely correlative nature, and we cannot differentiate between retained vs. acquired Lgr5 expression. Molecular profiling of Lgr5^(+)^/*LGR5*-expressing cell-derived tumors through the pathogenic pathway, including the precursors, could shed further light on the tumorigenic pathway. Lgr5^(+)^/*LGR5*-expressing cell-derived tumors may not be restricted to a specific molecular subtype, and CIN-type tumors are unlikely to be restricted to Lgr5^(+)^/*LGR5*-expressing cells as their sole cell-of-origin. The hierarchy of the stem/progenitor cells in the stomach await further clarification, and we await identification of other candidate cells-of-origin and to assess the impact of genetic manipulation of other candidate cells.

## Conclusions

Our results show that Lgr5 expression is dynamic in gastric/GEJ adenocarcinoma and heterogeneous across the several disease attributes. We postulate that this may reflect “retained stemness” in the form of Lgr5^High^-GSC signature that appears to be associated with better survival.

## Supplementary information

**Additional file 1.**

**Additional file 2.**

**Additional file 3.**

**Additional file 4.**

## Data Availability

The datasets used and/or analysed during this study are available from the corresponding author on reasonable request.

## Abbreviations

CIN: Chromosomal instability
CSC: Cancer stem cell
EBER: EBV-encoded small RNA
EBV: Ebstein-Barr Virus
GEJ: Gastroesophageal junction
GS: Genomically stable
IHC: Immunohistochemistry
GSC: Gastric stem cell
MSC: Mesenchymal stem cell
MSI: Microsatellite instability
MMR: Mismatch repair
OS: Overall survival
SMH: St. Michael’s Hospital
TCGA: The cancer genome atlas
TMA: Tissue microarray
