# Supplementary material for: Lgr5 and stem/progenitor gene expression in gastric/gastroesophageal junction carcinoma – significance of potentially retained stemness
Source: BMC Cancer. 2020 Sep 7;20:860. doi: 10.1186/s12885-020-07362-7 (PMC7487651; doi:10.1186/s12885-020-07362-7)
Supplement: Supplementary file 1 [file 12885_2020_7362_MOESM1_ESM.docx]

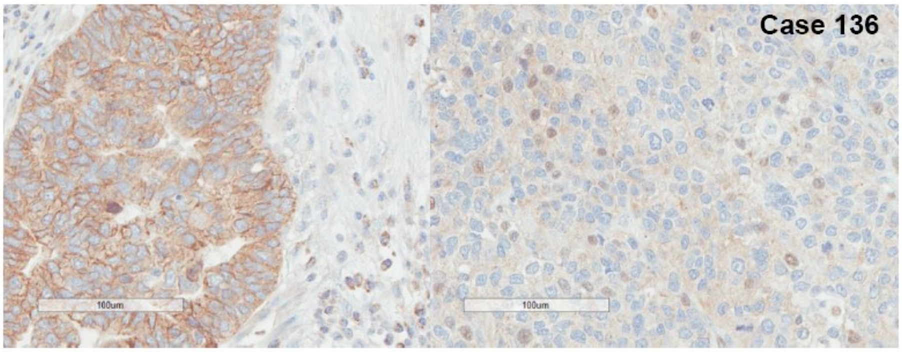

Supp. Fig. 1. Lgr5 expression pattern in the single case with both high cytoplasmic/menbranous Lgr5 (left) and nuclear expression (right). The area with nuclear Lgr5 expression was notable for more solid histology.
