# Supplementary material for: Lgr5 and stem/progenitor gene expression in gastric/gastroesophageal junction carcinoma – significance of potentially retained stemness
Source: BMC Cancer. 2020 Sep 7;20:860. doi: 10.1186/s12885-020-07362-7 (PMC7487651; doi:10.1186/s12885-020-07362-7)
Supplement: Supplementary file 2 [file 12885_2020_7362_MOESM2_ESM.docx]

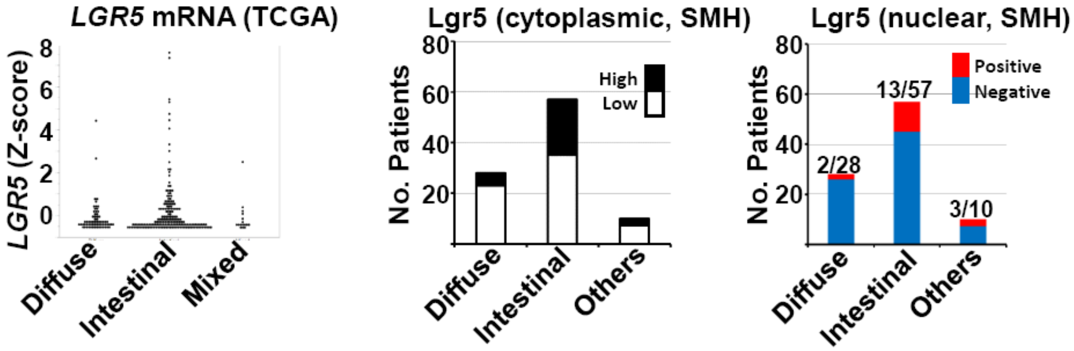

Supp. Fig. 2. Comparison of *LGR5* mRNA (TCGA cohort)/Lgr5 protein (SMH TMA) with respect to the indicated histology types.
