# Supplementary material for: Lgr5 and stem/progenitor gene expression in gastric/gastroesophageal junction carcinoma – significance of potentially retained stemness
Source: BMC Cancer. 2020 Sep 7;20:860. doi: 10.1186/s12885-020-07362-7 (PMC7487651; doi:10.1186/s12885-020-07362-7)
Supplement: Supplementary file 3 [file 12885_2020_7362_MOESM3_ESM.docx]

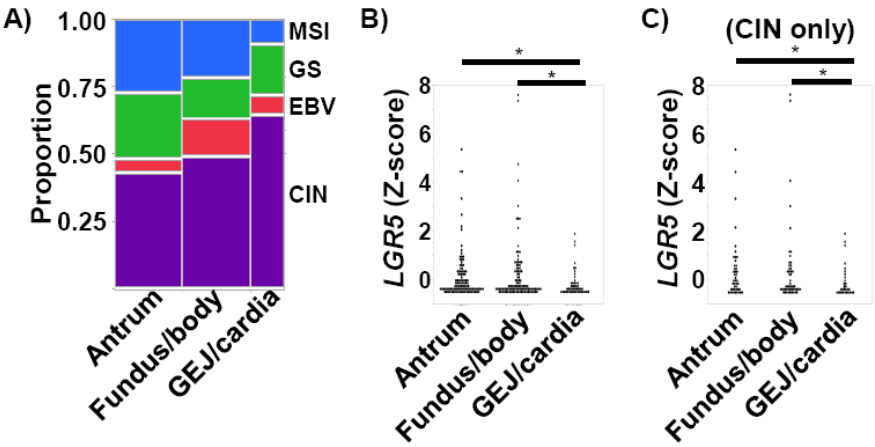

Supp. Fig. 3. A) Mosaic diagram showing the distribution of the different molecular subtypes by anatomical location in the TCGA cohort. B) *LGR5* mRNA levels in the TCGA cohort by anatomical location of tumors, with C) focusing on the CIN molecular subtypes.
