# Supplementary material for: Lgr5 and stem/progenitor gene expression in gastric/gastroesophageal junction carcinoma – significance of potentially retained stemness
Source: BMC Cancer. 2020 Sep 7;20:860. doi: 10.1186/s12885-020-07362-7 (PMC7487651; doi:10.1186/s12885-020-07362-7)
Supplement: Supplementary file 4 [file 12885_2020_7362_MOESM4_ESM.docx]

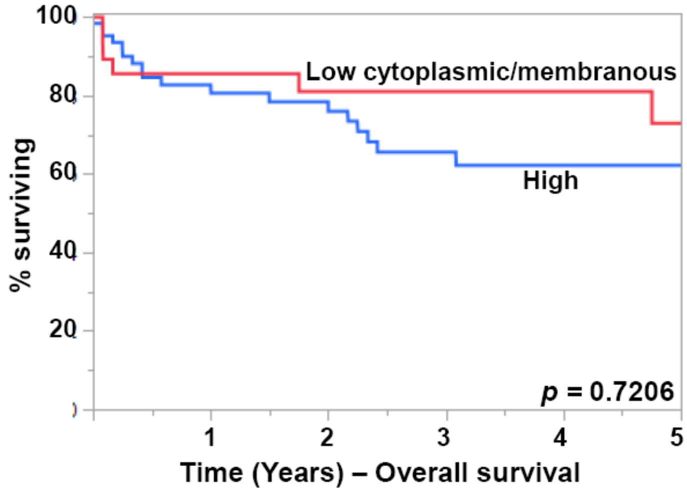

Supp. Fig. 4. Overall survival in the St. Michael's cohort, stratified by cytoplasmic/membranous Lgr5 expression levels.
